# Characterization of a human coagulation factor Xa-binding site on *Viperidae *snake venom phospholipases A_2 _by affinity binding studies and molecular bioinformatics

**DOI:** 10.1186/1472-6807-7-82

**Published:** 2007-12-06

**Authors:** Grazyna Faure, Veerabasappa T Gowda, Rachid C Maroun

**Affiliations:** 1 Unité d'Immunologie Structurale, Institut Pasteur, 25 rue du Dr. Roux, 75724 Paris Cedex 15, France; 2 University of Mysore, Department of Studies in Biochemistry, Manasagangothri, 570006 Mysore, India; 3 INSERM, Centre Paul Broca, 2-ter rue d'Alésia, 75014 Paris, France

## Abstract

**Background:**

The snake venom group IIA secreted phospholipases A_2 _(SVPLA_2_), present in the *Viperidae *snake family exhibit a wide range of toxic and pharmacological effects. They exert their different functions by catalyzing the hydrolysis of phospholipids (PL) at the membrane/water interface and by highly specific direct binding to: (i) presynaptic membrane-bound or intracellular receptors; (ii) natural PLA_2_-inhibitors from snake serum; and (iii) coagulation factors present in human blood.

**Results:**

Using surface plasmon resonance (SPR) protein-protein interaction measurements and an *in vitro *biological test of inhibition of prothrombinase activity, we identify a number of *Viperidae *venom SVPLA_2_s that inhibit blood coagulation through direct binding to human blood coagulation factor Xa (FXa) via a non-catalytic, PL-independent mechanism. We classify the SVPLA_2_s in four groups, depending on the strength of their binding.

Molecular electrostatic potentials calculated at the surface of 3D homology-modeling models show a correlation with inhibition of prothrombinase activity. In addition, molecular docking simulations between SVPLA_2 _and FXa guided by the experimental data identify the potential FXa binding site on the SVPLA_2_s. This site is composed of the following regions: helices A and B, the Ca^2+ ^loop, the helix C-β-wing loop, and the C-terminal fragment. Some of the SVPLA_2 _binding site residues belong also to the interfacial binding site (IBS). The interface in FXa involves both, the light and heavy chains.

**Conclusion:**

We have experimentally identified several strong FXa-binding SVPLA_2_s that disrupt the function of the coagulation cascade by interacting with FXa by the non-catalytic PL-independent mechanism. By theoretical methods we mapped the interaction sites on both, the SVPLA_2_s and FXa. Our findings may lead to the design of novel, non-competitive FXa inhibitors.

## Background

Haemostasis (vasoconstriction, platelet plug formation and blood clotting) is a defense mechanism that evolved to prevent the loss of blood after injury to the transporting vessels [1]. The intrinsic and the extrinsic alternate pathways initiate the blood clotting process. One of the common steps in both pathways of coagulation is the activation of coagulation factor X (FX) to factor Xa (FXa).

FXa circulates in plasma as the light and the heavy chains connected by a single disulfide linkage. The N-terminal region of the light chain (residues 1–39) is the Gla domain, rich in post-translationaly modified γ-carboxyglutamic acid, which interacts with the phospholipid (PL) membrane[2]. The Gla domain is followed by a short stack of hydrophobic residues (residues 40–45), and two epidermal growth factor-like repeats – the EGF-like 1 domain (residues 46–84) and the EGF-like 2 domain (residues 85–128). The heavy chain of FXa contains the catalytically active serine proteinase domain (254 amino acids, residues 16–269 in chymotrypsinogen numbering system). As a serine proteinase of the chymotrypsin family [3], FXa consists of two subdomains of antiparallel β-barrel structure each comprising a sheet of six strands and four helices. Residues His57, Asp102, and Ser195 (chymotrypsinogen numbering) form a catalytic triad at the active site cleft between the two subdomains[4]. The fold contains a number of solvent-exposed loops, which determine S1 and subsite preferences in structurally homologous enzymes of the family. To the north of the active site cleft in the canonical view are the 60- and 99-loops; to the west are the 174 – and the 217–225 loops, restricting access to the active site. The autolysis loop 149–151 occupies the southern boundary of the active site cleft. Adjacent to it is the 70-loop. To the east is the 37-loop. Loop 185–188 is associated with S1 preference[5,6]. Upon binding to FVa in the presence of Ca^2+ ^ions on negatively charged membrane PL at the cellular surface, the prothrombinase complex is formed, resulting in the accelerated conversion of FII (prothrombin) to FIIa (thrombin) by FXa [7,8]. Afterwards, thrombin converts fibrinogen into fibrin, consolidating the primary plug.

Secreted phospholipases A_2 _(sPLA_2_, EC 3. 1. 1. 4) are water-soluble interfacial enzymes that catalyze the hydrolysis of the 2-acyl groups in 3-*sn*-phosphoglycerides. The His48/Asp99 pair, the 26–34 calcium-binding loop and the 69-loop of residues 59–74 constitute the catalytic site. The calcium metal is a cofactor and its pocket is composed of Asp49 and the calcium-binding loop [9]. The Interfacial Binding Site (IBS) of several PLA_2_s has been located and is species- and enzyme class-specific [10-12]. In human non-pancreatic secreted group IIA phospholipase A_2 _(hsPLA_2_), the IBS is located in the "front" face of the enzyme [13,14]. It consists of a highly hydrophobic surface (Val3, Ala18, Leu19, Phe24, Phe70 and Tyr119) that surrounds the active site, and of hydrophilic residues (Arg7, Lys10, Glu16, and His6) (PDB code 1BBC; numbering system throughout as in Renetseder *et al. *[15]). Two other residues (Lys74 and Lys115) lie in the periphery of the IBS[13].

In addition to the esterase activities, sPLA_2_s are also specific ligands that interact with different targets, such as membrane-bound PLA_2 _receptors [16,17], anionic heparan sulfate proteoglycans (HSPG)[18,19], and with a cytoskeleton protein (vimentin)[20]. On the other hand, the soluble receptors of PLA_2_s, such as the natural inhibitors in the blood of snakes, the coagulation factors, and the PLA_2 _binding protein have been also well characterized[21,22]. Hence, it has become clear that PLA_2_s exert physiological and patho-physiological effects through protein-protein interaction and/or protein-PL interactions[22,23]. These protein-protein mechanisms are sometimes dependent on, and other times independent of their enzymatic activity, playing important roles in determining the specific function of sPLA_2_s [24-26]. *Viperidae *snake venoms contain several toxic group IIA PLA_2_s (SVPLA_2_), which may act as presynaptic neurotoxins[27] and may interfere with blood coagulation by possessing strong anticoagulant properties [28-40]. *Viperidae *SVPLA_2_s have high primary sequence identity with human group IIA PLA_2 _(hsPLA_2_; 30–60%) and overall structural homology[41,42]. Almost all anticoagulant snake venom PLA_2 _are basic proteins and may inhibit coagulation by several mechanisms. A first mechanism involves the hydrolysis and destruction of procoagulant PL [43-47]. CM-I and CM-II, illustrate this mechanism in which the group I PLA_2_ inhibits the extrinsic tenase complex [22,43], and does not bind to FXa. A second mechanism is based on the competition with clotting proteins for binding to the lipid surface due to the high affinity of PLA_2 _toward PL the "antagonist effect" [28,48][49]. A third mechanism is a non-enzymatic, PL-independent mechanism in which the PLA_2 _interacts with FXa, inhibits the prothrombinase complex by preventing formation of the FXa/FVa complex and introduces a lag time in the generation of thrombin[22,32,34,35,39,50,51]. In addition, some snake venom PLA_2_s show a combination of enzymatic and non-enzymatic mechanisms, like the basic sPLA_2 _isoform CM-IV from *Naja nigricollis (Elapidae) *venom[22,29].

The non-enzymatic inhibition mechanism of the prothrombinase complex was first demonstrated for CM-IV[32,34] and then for hsPLA_2 _[50]. The catalytically inactive His48Gln mutant of hsPLA_2 _possesses an identical anticoagulant effect and binds to FXa with the same kinetic constant as the wild-type enzyme, showing that in this mechanism the anticoagulant process is independent of the catalytic activity of the PLA_2 _[51].

Kini and Evans first proposed that a "pharmacological site," in addition to the catalytic site, could explain the specific biological anticoagulant activity of snake venom group I PLA_2_s[33]. This anticoagulant region consists of the basic exposed loop located in the region 55–70, and the beginning of the β-sheet on *Elapidae *group I PLA_2_s [52,53]. The proposed region is positively charged in strong anticoagulant enzymes, but negatively charged in weak and non-anticoagulant enzymes [33]. More recently, Kini proposed that weakly anticoagulant enzymes, which lack the anticoagulant region, fail to bind specifically to FXa in the coagulation cascade [22].

In spite of all the studies, the corresponding site in *Viperidae *SVPLA_2_s is not yet clearly established. In addition, no distinction is usually made between the mechanisms by which the anticoagulant potency is exerted by the PLA_2_s. It seems that only the mechanisms that imply participation of PL have been taken into account in the past.

Anticoagulant snake venom PLA_2 _represent a novel family of agents useful in identifying the sites of interaction of anticoagulants at the level of specific amino acid residues and thus have a potential in identifying new drug leads [54]. In order to characterize the FXa-binding site of SVPLA_2 _from the *Viperidae *family, we have focused on the non-enzymatic, PL-independent, anticoagulant mode of action. Previous work from the Unité des Venins (Institut Pasteur, Paris) had shown the involvement of basic residues located around the IBS of hsPLA_2 _[51], on one hand, and the possible involvement of the C-terminal and β-wing regions of AtxA[39] in binding to FXa, on the other. We were henceforth interested in determining whether similar or different amino acid patterns were present in *Viperidae *SVPLA_2_s. Using SPR and a physiological test of inhibition of prothrombinase activity, we identified SVPLA_2_s that formed complexes with FXa and determined the apparent affinity constants of the complexes. With this experimental information at hand, we applied sequence analysis, molecular bioinformatics and docking procedures in order to define the anticoagulant region of the PLA_2 _and the nature of the residues involved in the interaction with FXa.

The SVPLA_2_s tested in this study are: CBc and CBa_2_, two isoforms of the basic subunit of crotoxin (CTX), a non covalent, heterodimeric toxin from *Crotalus durissus terrificus *formed by the basic CB and the acidic CA subunits [55]; CA_2_, an isoform of the acidic subunit of CTX[56]; the acidic and basic subunits of the β-neurotoxin from *Pseudocerastes fieldi*[57] (CbI and CbII); isoform A of ammodytoxin from *Vipera ammodytes ammodytes*[58] (AtxA); the PLA_2 _from *Vipera berus berus*[59] (*Vbb*); Myotoxin II (inactive Asp49Lys mutant) [60] from *Bothrops asper*[61] (MtxII); PLA_2 _from *Daboia russelli pulchella *[62] (VRV-PLVIII); the basic PLA_2 _from *Agkistrodon halys pallas *[63] (*bAhp*); agkistrodotoxin from *Agkistrodon halys pallas*[64] (AGTX); PLA_2 _from *Crotalus atrox *[65] (*Catx*). We also tested human group IIA PLA_2_[66] (hsPLA_2_; Uniprot P14555; PDB 1BBC). The crystal structures of MtxII, VRV-PLVIII, *bAhp*, AGTX, *Catx *and hsPLA_2 _are available in the PDB, whereas we structure-modeled CBc, CBa_2_, CbI, CbII, AtxA and *Vbb*.

## Results

### Identification of SVPLA_2_s that bind to FXa and inhibit prothrombinase activity

As shown in Table 1, the SVPLA_2_s from *Viperidae *snake CBc and MtxII interact with FXa with very high affinity and constitute group VS (<K_d_^app^> 0.6 – 2 nM). These enzymes strongly inhibit the formation of the prothrombinase complex (IC_50 _1–3 nM). Under identical conditions, the <K_d _^app^> value determined for hsPLA_2 _is 14 nM. CBa_2_, AtxA and CbII have high affinity values and anticoagulant potency, and form group S (20–50 nM). A third group of SVPLA_2_, group M, is formed by *Vbb*_, _VRV-PLVIII, and *bAhp*. In this group, the affinity with FXa is smaller (<K_d_^app^> 400 – 830 nM) and the IC_50 _ranges from 90 to 130 nM. AGTX (the neutral PLA_2 _from *A. halys pallas (blomhoffii)*), reported previously as weakly anticoagulant in the presence of PL[38], does not interact with FXa and does not inhibit prothrombinase activity in the absence of PL. Neither CbI nor *Catx *inhibit prothrombinase activity in the absence of PL. These three SVPLA_2_s constitute group NB. Last, reduced and carboxymethylated CBc does not interact with FXa. Furthermore, we found a positive linear correlation between <K_d _^app^> and IC_50 _(R = 0.995) for the three groups of SVPLA_2 _that interact with FXa. Thus, the strongly anticoagulant SVPLA_2_s bind with high affinity to FXa, whereas the less efficient anticoagulant SVPLA_2 _possess low affinity for FXa. Also, basic SVPLA_2_s (8.35<pI<9.10) bound to FXa and inhibited prothrombinase activity, whereas those with acidic pIs (4.60–5.43) did not (Table 1).

**Table 1:** FXa binding kinetic parameters and effect on prothrombinase activity of *Viperidae *SVPLA_2_

**PLA_2_**	**<k_on_>(M^-1^s^-1^)**	**<k_off_> (s^-1^)**	**<K_d_^app^>[nM]**	**IC_50_[nM]**	pI*
**CBc**	(3.2 ± 0.2) × 10^5^	(1.6 ± 0.4) × 10^-4^	0.6 ± 0.3	0.7 ± 0.3	8.74
**MtxII**	(10.4 ± 0.3) × 10^6^	(1.78 ± 0.4) × 10^-2^	1.8 ± 0.9	3 ± 1	9.10
**CbII**	(4.2 ± 2.5) × 10^5^	(8.5 ± 2) × 10^-3^	20 ± 3	20 ± 4	8.96
**AtxA**	(2.2 ± 0.3) × 10^5^	(7 ± 1) × 10^-3^	30 ± 2	25 ± 5	8.35
**CBa_2_**	(2.9 ± 0.4) × 10^5^	(1.5 ± 0.2) × 10^-2^	52 ± 4	41 ± 5	8.74
**VRV-PLVIII**	(4.5 ± 0.4) × 10^4^	(2.6 ± 0.4) × 10^-2^	578 ± 15	130 ± 20	8.35
**b*Ahp***	(4 ± 1.5) × 10^4^	(1.6 ± 0.3) × 10^-2^	400 ± 20	90 ± 10	8.71
***Vbb***	(3 ± 0.5) × 10^4^	(2.5 ± 0.4) × 10^-2^	830 ± 15	90 ± 30	8.64
**AGTX**	NB	NB	NB	>10 000	5.43
**CA**	NB	NB	NB	>10 000	-
***Catx***	NB	NB	NB	>10 000	4.64
**CbI**	NB	NB	NB	>10 000	4.86
**Reduced and carboxymethylated CBc**	NB	NB	NB	nd	nd
**hsPLA_2_**	(2.0 ± 0.8) × 10^6^	(2.9 ± 1) × 10^-2^	14 ± 2	9 ± 2	9.38

*: estimated

NB: non-binding

Nd: not determined

<K_d_^app^> = <koff>/<kon>

The IC_50 _value corresponds to 50% inhibition of thrombin generation in the absence of PL for the different SVPLA_2_.

It is interesting to note that the isoenzymes CBc and CBa_2_, which differ by 8 amino acids (His1Ser, Ile18Val, Arg34Gln, Pro74Arg, Glu92Lys, Tyr115Asn, Gly116Glu, Gly128Glu) and associate with the acidic subunit CA to form two pharmacologically distinct classes of crotoxin complexes, present differences in toxicity, enzymatic activities and stability[67,68]. These two isoforms bind to FXa with different kinetics (Table 1). The average rate of dissociation constant <k_off_> for the FXa-CBa_2 _complex is about two orders of magnitude greater than that of the FXa-CBc complex, implying a more stable FXa-CBc complex (<K_d _^app^> 0.6 nM). Consequently, CBc strongly inhibits the prothrombinase complex (IC_50 _0.7 nM), whereas the inhibition by CBa_2 _is much weaker (IC_50 _41 nM). Indeed, we had observed in the past that the difference in stability between crotoxin isoforms was due only to the CB subunit [55,68].

**Figure 1:**
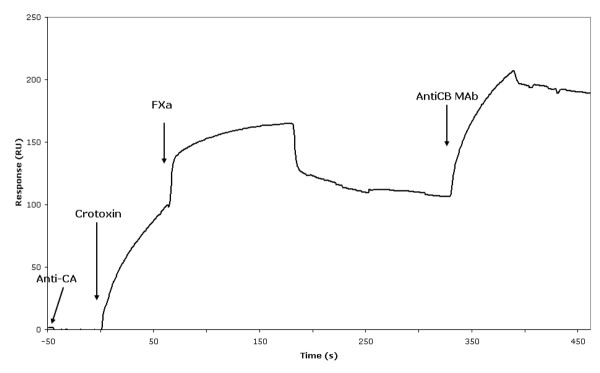
**The interaction between an isoform of CTX and FXa, as measured by SPR**. The anti-CA monoclonal antibody mAb A-73.13 [70] was covalently attached to the chip [71]. CTX (the CA_2_-CBc complex) was then captured via this mAb before injection of FXa (25 μg/ml). FXa bound to CTX, as seen in the rise of the resonance signal, showing that the CTX-FXa complex remained attached to the anti-CA mAb. After injection of a specific anti-CB MAb (B-32.13, 10 μg/ml), the signal increased further, confirming the presence of CB in the ternary CA-CB-FXa complex.

We also investigated by SPR the possibility of the formation of a ternary CA-CB-FXa complex. On one hand, CA binds to immobilized CB [69], whereas FXa does not interact with immobilized CB[23]. An anti-CA monoclonal antibody (mAb) A-73.13 [70] was covalently attached to the chip, as previously described [71]; CTX was then captured via this mAb before the injection of FXa (Fig. 1). FXa bound to CTX, as seen in the rise of the resonance signal. Fig. 1 also shows that after the injection of a specific anti-CB mAb (B32.13), the signal increased further, indicating the presence of CB on the chip and showing that the CB-FXa complex is stable and remained attached to CA.

### Sequence analysis, comparisons and consensus residues of the anticoagulant SVPLA_2_s

In order to identify those residues or sequence patterns that differ between members of the SVPLA_2_s, we obtained Weblogo plots of the four groups VS (CBc, MtxII), S (CbII, AtxA, CBa_2_), M (VRV-PLVIII, *bAhp*, *Vbb*) and NB (AGTX, *Catx*, CbI). Thereafter, we performed comparisons of all the sequences among themselves and of the four groups against each other.

**Figure 2:**
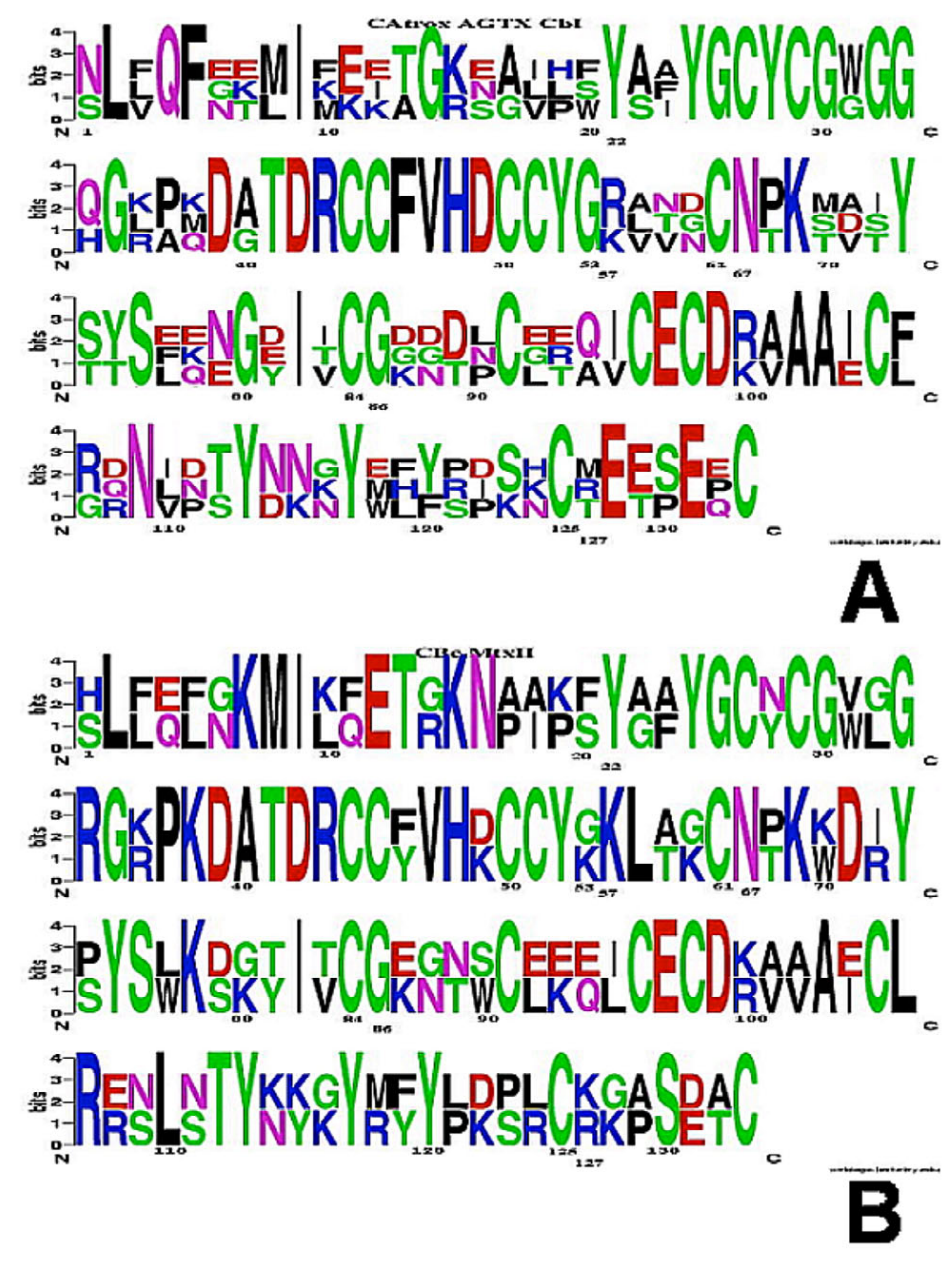
**Weblogo representation of the multiple sequence alignment of the SVPLA_2_s**. A. Group NB (AGTX, *Catx *and CbI). Each logo consists of stacks of symbols, one stack for each position in the sequence. The overall height of the stack indicates the sequence conservation at that position, while the height of symbols within the stack indicates the relative frequency of each amino acid at that position. The symbols in each stack are arranged by alphabetical order from top to bottom and do not follow the order in which the sequences were fed. Arg, His and Lys residues are in blue; Asp and Glu in red; Ala, Ile, Leu, Met, Phe, Pro, Trp, and Val in black; Gly, Cys, Ser, Thr, and Tyr, in green; Asn, Gln in purple. Renetseder numbering system for all PLA_2_s throughout this work [15]. B. Group VS (CBc and MtxII).

Considering only segments of three or more residues, we observed three conserved regions for AGTX, *Catx *and CbI of the NB group (Tyr25-Gly30, Thr41-Gly53 and Cys96-Asp99; Fig. 2A), and six for the CBc-MtxII pair of the VS group (Tyr25-Cys27, Gly33-Gly35, Pro37-Cys45, Cys50-Tyr52, Cys96-Asp99 and Cys105-Arg107; Fig. 2B). A striking difference between the two groups is the presence of the acidic residue Glu at position 128 in the C-terminal region of all SVPLA_2_s of the NB group, as opposed to a Lys or a Gly residue in the VS group. For the other groups, only CBa_2 _and *Vbb *contain a Glu at this position. With respect to the S group, the M group contains a conserved basic residue at position 115. Interestingly, the M group contains a conserved Lys in position 132 that is absent in the VS and NB groups. Group NB contains acidic side chains in the first strand of the β-wing and the adjacent β-turn (residues 75–82). For the VS and S groups, the tendency is towards a net balance of positive charges in the β-wing.

The NB group *Catx *presents three Glu and one Asp residue in the β-wing (residues 74–90); CbI presents two Asp residues. The β-wing of CBc of group VS contains two Lys residues; that of MtxII two Lys, one Asp and one Glu residues. For the S and M groups the tendency is towards net positive charges, except for *bAhp*, for which the total charge is neutral.

### Tertiary structures and molecular electrostatic potentials of SVPLA_2_s

**Figure 3:**
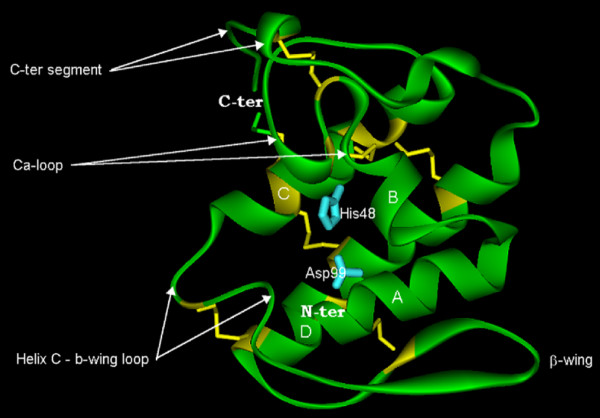
**Ribbon diagram of the molecular model of AtxA**. α-helices A, B, C and D, and the β-wing are labeled. The seven-disulfide bridges are in yellow sticks and the His48/Asp99 pair in cyan sticks.

We obtained 3D homology models of CBc, CBa_2_, CbI, CbII, AtxA and *Vbb*. The models show the canonical structural features of the PLA_2 _template molecules: a N-terminal α-helix (helix A), a short helix (helix B), a Ca^2+^-binding loop, a long α-helix (C), a loop preceding an anti-parallel two-stranded sheet (β-wing), a long α-helix (D), anti-parallel to helix C, and a C-terminal extended fragment. All seven disulfide-bridges are at their expected positions. Fig. 3 shows the molecular model of AtxA in the canonical ("front") face orientation, i.e., presenting the catalytic hydrophobic channel.

**Figure 4:**
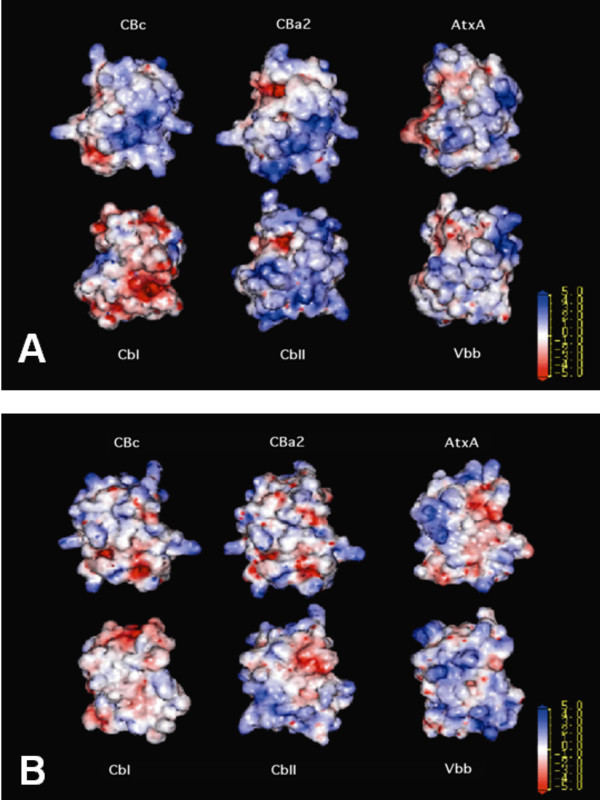
**MEP at the solvent accessible surface of the 3D molecular models of the *Viperidae *SVPLA_2_s**. A. The models correspond to CBc, CBa_2_, AtxA, CbI, CbII, and *Vbb*. Color codes correspond to MEP in kT/e units: blue, +5; red, -5; white, 0. Front view. B. Back view.

Fig. 4A shows the MEP calculated at the molecular surface of the modeled SVPLA_2_s in the canonical view. Fig. 4B shows the MEP on the "back" face. Qualitatively, we observe that the MEP is positive in the front face of strong and mild anticoagulants (CBc, CBa_2_, CbII, and AtxA); approximately neutral for the weak anticoagulant *Vbb*, and negative for CbI (Fig. 4A). The back face is largely devoid of positive potential, except for *Vbb *(Fig. 4B). This difference in MEP leads to an electrostatic asymmetry. We observe the same trend for the crystallized SVPLA_2 _(MtxII, VRV-PLVIII, *bAhp*, AGTX, *Catx *and hsPLA_2_; not shown).

### Molecular docking and intermolecular interfaces: mapping of the anticoagulant site and of the binding site on FXa

**Figure 5:**
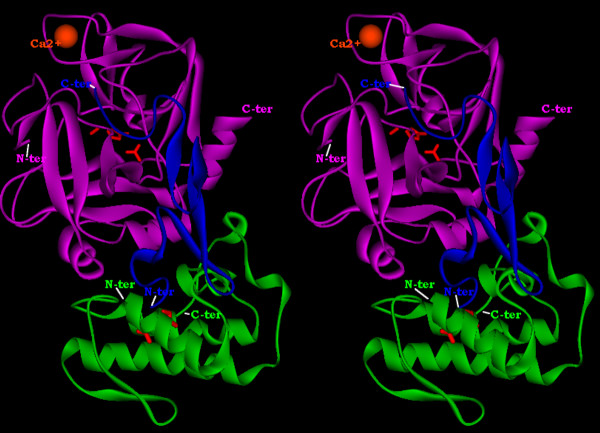
**Ribbon representation of the 3D molecular model of the complex between AtxA and the light and heavy chains of FXa**. Crossed-eye stereo ribbon representation, with AtxA in green, the light and heavy chains of FXa in blue and purple, respectively. Catalytic site residues are red sticks for both AtxA (His48, Asp99) and FXa (His57, Ser102, Asp195). The metal Ca^2+ ^ion of FXa is depicted as an orange sphere. The N- and C-ter of the AtxA, FXa's heavy chain and FXa's light chain are indicated.

**Figure 6:**
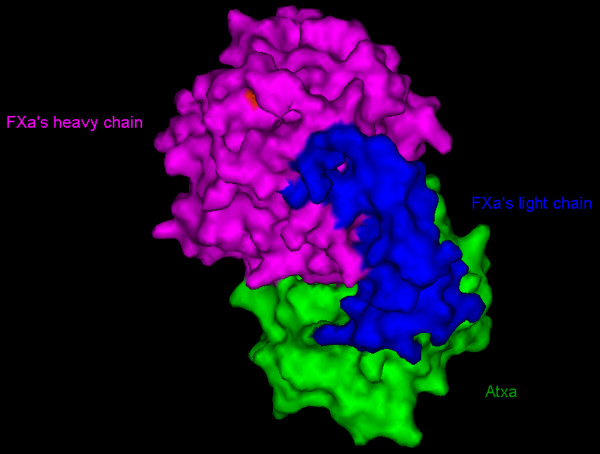
**Solvent-accessible surface representation of the 3D molecular model of the complex between AtxA and the light and heavy chains of FXa**. Color coding as in Fig. 5.

The output generated by the docking program PatchDock showed many complexes in contact with one of the binding site regions. Only the AtxA-FXa candidate complex (rank number 7) showed the best compatibility with the binding site derived from the mutagenesis data[39] (in this complex, position 150, which shows a Glu -> Arg sequence difference between the purchased FXa and the crystal structure (PDB entry 2BOH) is neither at the light/heavy chains interface nor at the AtxA/FXa interface). Several residues belonging to the two regions identified by the mutagenesis of AtxA and defining part of the binding site (the "front" strand of the β-wing and the C-terminal fragment) are at the interface in this complex. In it, the relative orientation of FXa positions the N-terminus of the EGF-like 2 domain of the light chain of FXa towards the β-wing of AtxA, allowing the 1-domain (not visible in the X-ray structure) to enter in interaction with the β-wing and thus contact the remaining residues of the front edge of the β-wing. Fig. 5 shows the ribbon representation of the final complex between AtxA and the light and heavy chains of FXa. Fig. 6 shows the same complex in the form of a solvent-accessible surface. In both representations, the SVPLA_2 _is in the "classical" orientation, i.e. that of Fig. 4A. Overall, we observe just small conformational changes after complex formation.

For the FXa strong-binding SVPLA_2_s CBc, MtxII, CbII and CBa_2_, the complexes that ranked 18, 10, 20 and 17, respectively, showed the same binding mode as the AtxA-FXa complex, i.e., the same relative orientation of FXa with respect to the SVPLA_2_. As measured by the FIT function of Pymol, these complexes showed Cα RMSD's with respect to the reference AtxA-FXa complex of 3.4, 2.2, 5.1 and 4.8 Å, respectively.

**Figure 7:**
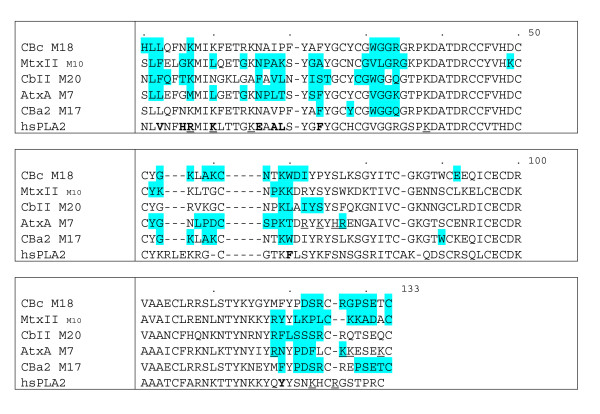
***Viperidae SVPLA*_2 _interface amino acid residues. **Interface amino acid residues of the SVPLA_2 _complexes with FXa (CBc, MtxII, CbII, AtxA and CBa_2_). Underlined characters denote residues identified by mutagenesis to be critical for binding to FXa and inhibition of prothrombinase activity [39, 51]. Bold characters denote residues defining the IBS of hsPLA_2 _[13, 14]. Cyan-shadowed characters denote residues found at the interface of the selected complexes. The alignment reflects Renetseder's numbering system.

**Figure 8:**
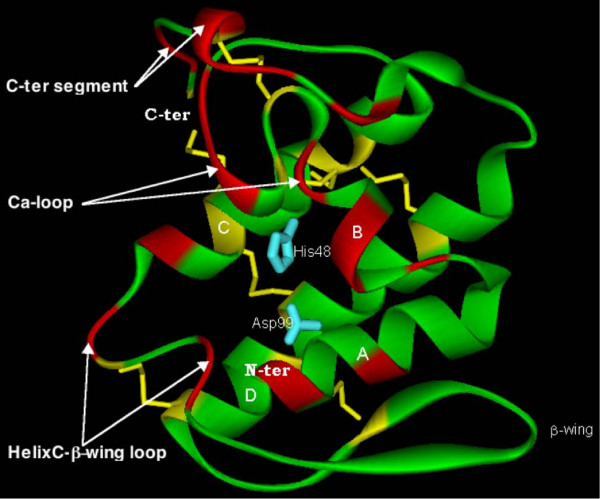
**Ribbon diagram of the molecular model of AtxA showing the identified interface regions. **The identified interface regions in SVPLA_2_s from Fig. 7 and made up of consensus positions 2, 3, 7 (helix A); 16; 18, 19 (helix B); 23, 24; 31–34 (Ca^2+ ^loop); 53, 59, 60, 69, 70 (helix C-β-wing loop); and 118, 119, 121–124, 129–131, 133 (C-terminal segment), are in red (see Results section).

The percentage of surface residues at the interface of the complexes is of 24–36% for the five SVPLA_2_s and of 20–32% for FXa. The interface area for the five SVPLA_2_s is in the 860–1700 Å^2 ^range and 915–1470 Å^2 ^for FXa. The interface area of the complexes varies from 1610 to 1930 Å^2^. The values obtained for the solvent accessible area buried in the five characterized complexes fall between the small and the large interfaces categories in a study of 362 protein-protein interfaces [72]. Fig. 7 shows the mapping of the SVPLA_2_-FXa interaction surface of the complexes between the five SVPLA_2_s and FXa. The shadowed residues in that figure are those at the interface of the AtxA-FXa complex satisfying the mutagenesis data, and at the interface of the complexes of the other four SVPLA_2_s that show the same complexation mode as the AtxA-FXa complex. The consensus residues that participate to FXa binding are: solvent-exposed parts of helix A (positions 2, 3, 7) and helix B (positions 18, 19); positions 16, 23 and 24; a part of the Ca^2+ ^loop (positions 31–34); a part of the 69-loop (between helix C and the β-wing; positions 53, 59, 60, 69 and 70); and the C-terminal segment (positions 118, 119, 121–124, 129–131, 133). By taking into account the type of atom-atom contacts as defined by Sobolev *et al.*[73], 36.5% of the contacts are hydrophobic at the AtxA-FXa light chain interface, as compared to 26.4% at the AtxA-FXa heavy chain interface. Overall, 29.7% of the contacts are hydrophobic at the AtxA-FXa interface. These interface regions are identified separately by the protein-protein interface prediction server PPI-Pred. For example, for AtxA, the highest score PPI-Pred patch, which represents the most probable protein-protein binding site, includes helices A and B, the Ca^2+^-loop, part of the 69-loop, two residues from the front strand of the β-wing, and the four C-terminal residues (results not shown). Fig. 8 shows the regions mapped by docking simulations in the modeled structure of complexed AtxA.

**Figure 9:**
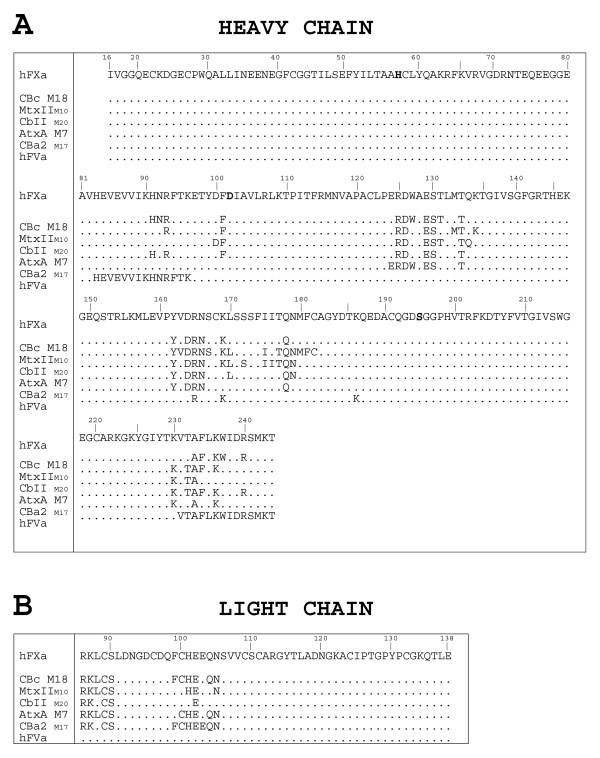
**FXa interface amino acid residues**. A. Amino acid residues of the heavy chain of FXa at the interface of the selected docked complexes for each SVPLA_2_. The sequence of FXa shows only residues detected in the X-ray experiment. The last line shows the experimentally reported residues of FXa involved in the binding to FVa [2, 78, 79, 83]. We use Renetseder's notation for the SVPLA_2_s and chymotrypsinogen notation for FXa. The sequences of CBc, AtxA, CbII and CBa_2 _were aligned with respect to MtxII. B. Amino acid residues of the light chain of FXa at the interface of the selected docked complexes for each SVPLA_2_.

**Figure 10:**
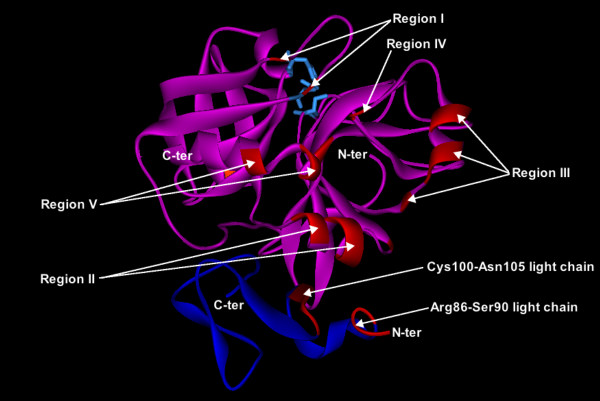
**Ribbon diagram of the crystal structure of FXa (PDB 2BOH) showing the identified interface regions**. The figure highlights in red the regions corresponding to the identified interface residues from Fig. 9A and B. In the light chain: Arg86-Ser90 and Cys100-Asn105. In the heavy chain: Region I: Arg93; Phe101 (from the 99-loop). Region II: Arg125; Asp126; Glu129-Ser130; Thr134. Region III: Tyr162; Asp164-Asn166; Lys169; Leu170. Region IV: Gln178 and Asn179 (from the 174-loop). Region V: Lys230, Thr232, Ala233, Phe234 and Lys236 (residues from the C-terminal helix). Only residues present three or more times in the same column in the sequences are included. Light chain is in blue, heavy chain in purple. FXa is rotated 90° in a clockwise sense about its vertical axes with respect to Fig. 5. The terminal ends of FXa's chains are labeled. The catalytic triad is represented as cyan sticks.

The first line of Fig. 9A and 9B show the amino acid sequences of the parts of the heavy and light chains of the crystal structure of FXa (PDB 2BOH), detected at the surface respectively. The heavy chain (Fig. 9A) includes the serine proteinase catalytic domain of FXa (catalytic-site residues in bold), and the light chain (Fig. 9B) includes the EGF-like 2 domain. The following lines in Fig. 9A and 9B show the FXa residues at the interface of the docked FXa-SVPLA_2 _complexes (residues appearing three or more times). The regions of FXa at the interface with CBc, MtxII, CbII, AtxA and CBa_2 _in the resulting docked complexes include, in the light chain (Fig. 9B), the N-terminal region of the EGF-like 2 domain: Arg86-Ser90 and Cys100-Glu102, Gln104, Asn105. In the heavy chain (Fig. 9A), they include the following regions. Region I: Arg93; Phe101 (from the 99-loop). Region II: Arg125; Asp126; Glu129-Ser130; Thr134. Region III: Tyr162; Asp164-Asn166; Lys169; Leu170. Region IV: Gln178 and Asn179 (from the 174-loop). Region V: Lys230, Thr232, Ala233, Phe234 and Lys236 (all residues from the C-terminal helix). Fig. 10 shows these regions in the 3D model of complexed FXa. Some of these residues belong to loops surrounding the catalytic-site cleft.

**Figure 11:**
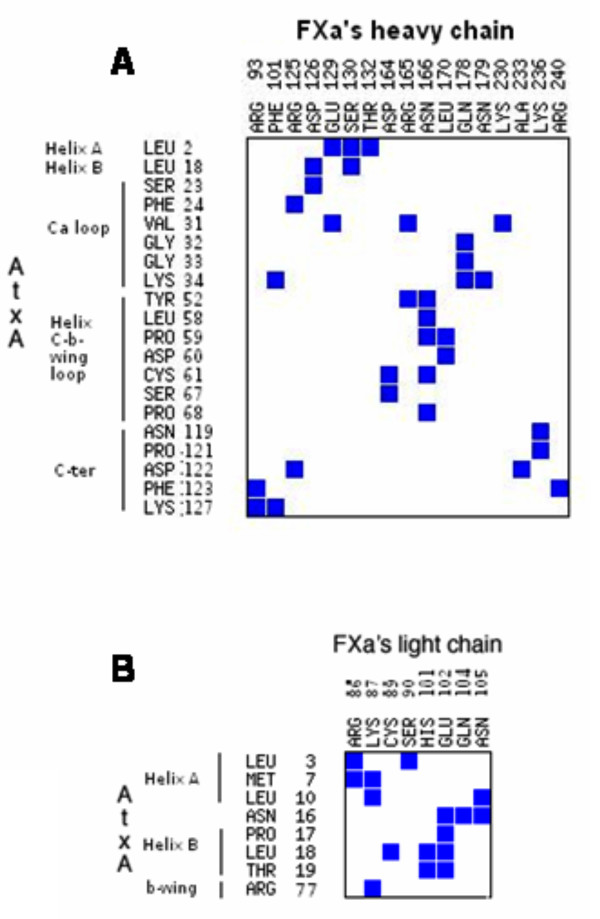
**Contact map for the AtxA-FXa complex**. A. Heavy-chain FXa residues. Only residues for which the contact area is equal or greater than 10 Å^2^ are shown.B. Light-chain FXa residues.

We show in Fig. 11A and 11B the intermolecular residue contacts for the AtxA-FXa interface for which the contact area is equal to or greater than 10 Å^2^. We observe from this map that the heavy chain of FXa interacts with the following regions of the PLA_2_: a portion of the Ca^2+ ^loop, the helix C-β-wing loop, and the C-terminal segment (including Lys127, which has an effect in the binding to FXa) (Fig. 11A). The light chain of FXa establishes intermolecular contacts with the remaining regions: helices A and B, and the β-wing (Arg77) of AtxA (Fig. 11B). From the CSU analysis of interfaces, it follows that the contacts between SVPLA_2 _and FXa are of diverse nature -aromatic-aromatic, hydrophobic-hydrophobic, salt bridges and H-bonds. There are also destabilizing hydrophobic-hydrophilic interactions. Few of the contacts take place between same-nature residues, like Lys127 from AtxA and Arg93 from FXa's heavy chain (Fig. 11A). These contacts are between non-polar atoms or between non-polar and polar atoms of side chains at the surface of the molecules, where the presence of the aqueous solvent can buffer the electrostatic interactions.

## Discussion

### Existence of different anticoagulant mechanisms

The classification of the anticoagulant potency (strong, weak, non-anticoagulant) of a SVPLA_2 _depends on the anticoagulant assay used and on whether this assay leads to establishing the mechanism of action. Usually, the recalcification time assay, one of the simplest assays, is used. This method is very sensitive to the lipid levels in the plasma but does not show whether the mechanism is PL-independent or not. Our results deal with the interaction of anticoagulant PLA_2_s with FXa through the non-catalytic, PL-independent mechanism of action and are to be compared to studies performed under the same conditions. As mentioned before, only three PLA_2_s -CM-IV of *N. nigricolis*, AtxA of *V. ammodytes ammodytes*, and hsPLA_2_- have been studied under these conditions. In this work, we complete these studies by testing several other SVPLA_2 _from *Viperidae *venom. Our results clearly show that CBc and MtxII interact with FXa with very high affinity and strongly inhibit the formation of the prothrombinase complex. CbII, AtxA and CBa_2 _possess good affinity for FXa and good anticoagulant potency. VRV-PLVIII, *bAhp *and *Vbb *possess weak affinity for FXa and show weaker inhibition of prothrombinase activity. Some of these enzymes (CB, AtxA) also inhibit prothrombinase activity in the presence of PL (not shown). AGTX, the CA subunit of CTX, *Catx *and CbI do not interact with FXa and do not inhibit prothrombinase activity in the absence of PL. Therefore, AGTX, described previously as a weakly anticoagulant PLA_2 _[38], and VRV-PLVIII, described previously as strongly anticoagulant [74], appear to inhibit blood coagulation through different mechanisms.

### Search for an anticoagulant site

#### Possible location of an anticoagulant site common to *Viperidae* SVPLA_2_s that interact directly with FXa

Reduced and carboxymethylated CBc did not interact with FXa (Table 1), suggesting that the SVPLA_2 _needs to adopt the proper conformation for the interaction to take place. In agreement and in the context of the PL-independent anticoagulant mechanism, our combined theoretical and experimental approach highlights the presence of an anticoagulant region composed of amino acid residues that come together in space to constitute a conformational epitope situated in the "front" face of the SVPLA_2_s. These are the solvent-exposed parts of helix A, helix B, the Ca^2+ ^loop, the helix C-β-wing loop, the front strand of the β-wing, and the C-terminal segment of the PLA_2 _(Fig. 8). Of course, the detailed distribution and composition of those residues varies for each of the SVPLA_2_s.

Carredano *et al.*[52] determined the 3D structure of group II A monomeric PLA_2 _RVV-VD from *Vipera r. russelli *(PDB 1VIP), described as a strongly anticoagulant SVPLA_2_. The authors proposed a site responsible for the strong anticoagulant properties of the toxin, consisting of Glu53, together with a positively charged ridge of non H-bonded lysine residues free for intermolecular interactions in the 53–70 region (Lys60 in RVV-VD and in CBa_2_). On another hand, Zhao *et al.*[75] suggested that residues Trp70 and Glu53 in *bAhp *might play an important role in the anticoagulant activity of the basic SVPLA_2_s. The study of Zhong *et al.*[76], who tested the anticoagulant potency of *bAhp *mutants, revealed that the Glu53Gly and Trp70Met mutants lost their effects on blood clotting, while Thr56Lys and Asp67Lys had enhanced activity. The reported residues fall in the 53–70 interface region detected in our docked complexes of the strong FXa binders CBc, MtxII, CbII, AtxA and CBa_2 _(Fig. 8). The possible contribution of Trp70 to the strong anticoagulant activity of PLA_2_s has also been proposed elsewhere [48]. Nevertheless, the anticoagulant region cannot be localized solely to the 53–70 segment, since enzymes that bind weakly or not at all to FXa contain also basic residues in this region.

The natural mutants CBc and CBa_2 _present two Gly -> Glu mutations in the C-terminal region (Gly116Glu, Gly128Glu) leading to increases in the IC_50 _values for inhibition of prothrombinase activity of CBa_2 _with respect to CBc. This is consistent with the results of our sequence comparison analysis, in which we detect the acidic residue at position 128 as characteristic of the NB group, and with the docking results that point to this region as being at the interface of the complexes.

On the other hand, we localized in the crystal structure of hsPLA_2 _(PDB 1BBC) the mutations that showed the major effects in the inhibition of prothrombinase activity and FXa-binding kinetic parameters [51]. Residues Arg7, Lys10 and Lys16 (helix A) are exposed to the solvent and form a cluster. Residue Lys38 (loop on N-terminus of helix B), and residues Lys123 and Arg126 form another cluster (underlined residues Fig. 7). As expected, the two clusters are situated on the front face and are oriented 180° about this convex surface. They act cooperatively in the binding to FXa. Lys86 carries with it an effect on IC_50_, but is in the end of the second strand of the β-wing ("back" face) of hsPLA_2_, indicating that its effect on IC_50 _is not due to the residue being at the interface. No effects are reported dealing with the Ca^2+ ^loop and the front strand of the β-wing does not appear in hsPLA_2 _(Fig. 7); however, to our knowledge, these regions have not yet been probed.

Lastly, our experimental data suggest that the CB-FXa interaction site is different from the CB-CA interface and show that the interaction between FXa and CTX proceeds through a transient ternary (CA, CB, FXa) complex (Fig. 1).

### The FXa binding region of PLA_2 _involves also hydrophobic residues

hsPLA_2 _contains an unusually large number of prominent cationic patches on its molecular surface, some of which lie on the putative IBS [14], in contrast to bovine pancreatic PLA_2 _and the SVPLA_2 _from *Agkistrodon p. piscivorus*, which display only a limited number of such patches [10]. Given the charged nature of the residues critical for binding hsPLA_2 _and AtxA to FXa, it is clear that electrostatic interactions play a role in the binding. Indeed, the electrostatic asymmetry showed by the MEP calculations must be enhanced by the presence of the essential Ca^2+ ^cofactor ion [77] and may be at the origin of the increased affinity of hsPLA_2 _for FXa in the presence of Ca^2+ ^[50]. Thus, long distance electrostatic forces operating at the molecular surface are important and may optimally orient the molecules before binding to FXa. However, electrostatic interactions might not exclusively drive binding to FXa by the *Viperidae *SVPLA_2_s, as in hsPLA_2_. Indeed, in addition to basic residues, hydrophobic and aromatic residues play also key roles in optimizing the interaction between SVPLA_2_s and FXa, given that a ring of hydrophobic residues surrounds the opening to the catalytic site cavity. Thus, for AtxA, many hydrophobic residues (Leu2, 3, 10, 18 and 58; Pro17, 59, 68 and 121; Val31), most of which are located in the N-terminal region, and several aromatic residues (Phe24 and 123; Tyr52) are part of the surface presented to FXa (Fig. 7). Lastly, even though a Phe24Ala mutation in AtxA was not found to change the FXa-binding kinetic parameters [39], we emphasize the need to probe other residues belonging to the hydrophobic ring.

### The PLA_2 _binding region of FXa involves both, the light and heavy chains but not the catalytic site

On our results, the catalytic site of FXa is free and not involved in the interface, in agreement with the conservation of the serine-proteinase catalytic activity after binding by SVPLA_2 _[51]. Our AtxA-FXa complex shows that one region of the light chain of FXa is involved in the binding to the SVPLA_2 _and that the N-terminus of the EGF-like 2-domain is pointing south (Fig. 5). Therefore, the EGF-like 1-domain, not visible in the crystal structure of FXa, can interact with the front edge of the β-wing of the PLA_2_. The Gla domain of FXa (bound to the N-terminus of the EGF-like 1-domain), is well beyond reach in space and not in contact with the PLA_2_. These two features are in agreement with experimental and biochemical data which support the conclusion that the Gla domain is needed rather for insertion into the PL membrane [51], and with a model of the entire FXa. Indeed, based on the crystal structure of the Gla domain of bovine prothrombin and the NMR coordinates of the bovine FX EGF1 domain, Bajaj and coworkers [2] proposed a model structure for the entire FXa molecule, based upon the crystal structure of porcine FIXa. The EGF domains in the model are oriented south and thus capable of establishing contacts with the β-wing of the PLA_2_.

Based on the ability of synthetic peptides from FXa to inhibit FXa-induced clotting, a number of authors have reported the FVa binding sites on FXa. The last line of Fig. 9A shows the experimentally reported residues of the heavy chain of FXa incontrovertibly involved in the binding to FVa. These residues are: FXa(His83-Lys96) [78]; FXa(Arg165, Lys169) [79-81]; the 185–189 loop, i.e., FXa(Lys186) [82]; FXa(Val231-Thr244) [83]; and FXa(Arg240) [81]. Three of four of the segments of the catalytic domain of FXa identified to interact with FVa overlap with those identified by us to interact with the *Viperidae *SVPLA_2_s, namely the segment about Lys90, the 162-loop, and the C-terminal part, about Lys237 (Fig. 9A). This suggests that several residues are shared by both, the SVPLA_2_-FXa and the FVa-FXa binding interfaces. Moreover, the experimentally identified FXa heavy chain residues involved in binding to FVa are located on the same 3D face of FXa, as in our SVPLA_2_-FXa complexes.

Arni and coworkers have recently reported the crystal structures of human Gla domainless FXa complexed with two small anticoagulant proteins from a hematophagous nematode [84,85]. The determined exosite from those complexes involves residues from one of the strands of the N-terminal seven-stranded β-barrel (strand β6, residues 80–93) and from the short C-terminal α-helix (residues 233–243) of the catalytic subunit of FXa. Several of these residues fall in regions I and V mapped in our complexes with the SVPLA_2_s (Fig. 9A and 10).

### Several IBS residues are part of the PL-independent anticoagulant site of SVPLA_2_

By combining the residues defining the IBS [13,14] with the site-directed mutagenesis experiments for probing the basic residues of hsPLA_2 _involved in binding to FXa[51], we deduce that IBS residues Arg7 and Lys10 bind to FXa.

By homology with hsPLA_2_, the presumed IBS amino acid residues for AtxA are Leu3, Leu18, Phe24, Lys74 and Tyr113. On one hand, Lys74, experimentally found to be critical for binding of AtxA to FXa [39], belongs to the IBS. On the other hand, our simulations indicate that IBS residues Leu3, Leu18 and Phe24 are in contact with FXa. In conclusion, several IBS residues are part of the PL-independent anticoagulant site and participate in formation of the complex with FXa.

### SVPLA_2 _are multifunctional proteins with multiple pharmacological sites

The *Viperidae *SVPLA_2_s studied here are multifunctional proteins, raising the possibility of overlapping or multiple pharmacological sites distinct from the catalytic site[86]. One team[52] has suggested that the neurotoxic site of group II neurotoxic enzymes overlaps with the anticoagulant region. Thus, from the mutants used to test the anticoagulant potency [39] and the neurotoxic function [87] of AtxA, it appears that several residues in the C-terminus are clearly shared by both functions. The overlapping of pharmacological sites is most easily understood in terms of the small size of this family of proteins.

## Conclusion

In this paper, we concentrated our efforts on identifying the anticoagulant *Viperidae *SVPLA_2_s that inhibit blood coagulation via a non-enzymatic, PL-independent mechanism through direct binding to human FXa. Using SPR technology, we showed that CBc and MtxII bind to FXa with the highest affinity and inhibit strongly the prothrombinase complex, whereas CbII, AtxA and CBa_2 _bind with good, but lesser affinity.

Of the eight mutations that differentiate CBc from CBa_2_, Arg34Gln in the Ca^2+ ^loop and Gly128Glu in the C-terminal segment fall in our identified interface regions; Gly116Glu is just borderline to the C-terminal fragment. Thus, the disappearance of a positive charge and the appearance of two negative charges in this region account for the loss of affinity of CBa_2 _for FXa with respect to CBc. This is consistent with our consensus sequence analysis, which had associated the presence of Glu at position 128 to a decrease in affinity.

The molecular electrostatic potential we calculate at the surface of the 3D molecular models shows a correlation with the anticoagulant potency of the SVPLA_2_s. However, since not all basic PLA_2 _are strong anticoagulants [44], the basic character of the PLA_2 _seems to be a necessary but not sufficient condition for its anticoagulant potency.

Mapping of the FXa-interface zone in the 3D structures of the SVPLA_2_s by binding-site directed docking simulations, allowed us to detect several FXa-binding regions that come together to form a conformational epitope on the "front" surface of the SVPLA_2_s. One of the regions maps to the 53–70 segment, proposed in the past to be the anticoagulant region. According to our findings, this region is to be extended, on one hand, to helices A and B and to the "front" strand of the β-wing, and to the Ca^2+ ^loop and the C-terminal direction, on the other. The FXa interface forms a novel exosite that involves both, the light and heavy chains.

Our work epitomizes the use of binding affinity and mutational experimental data in guiding molecular docking simulations by indicating which species associate and then outlining the possible interacting surface patches.

Finally, there has been intense interest in the development of FXa inhibitors for the treatment of thrombotic diseases. Anticoagulant *Viperidae *SVPLA_2_s, which interact with FXa via a non-catalytic, PL-independent mechanism, represent a novel family of selective FXa inhibitors. Structural information on the binding of these PLA_2 _to FXa should be useful in the 3D structure-based design of therapeutic agents. The synthesis of peptides or peptidomimetics derived from our mapped regions could lead to the development of new antithrombotic molecules capable of delaying *in vivo *the activation stage of the prothrombinase complex and to their use as supplementary agents in antithrombotic therapy.

## Methods

Reagents including Sensor Chips CM5, surfactant P20, the amine coupling kit containing *N*-hydroxysuccinimide (NHS), *N*-ethyl-*N'*-(3-diethylaminopropyl)carbodiimide (EDC) and ethanolamine hydrochloride were supplied by Biacore (Biacore AB, Uppsala, Sweden). All other chemicals and solvents of the highest available purity were obtained from either Merck A.G. (Darmstad, Germany), Prolabo (Paris, France) or Sigma Co (St. Louis, MO, USA). CbI and CbII from *Pseudocerastes fieldi *venom were supplied by Dr. A. Bdolah (Dept. of Zoology, Tel Aviv University, Israel) [88]. AtxA from *Vipera ammodytes ammodytes *was purchased from Latoxan. Recombinant hsPLA_2 _was produced in our laboratory as described previously[89]. Isoforms of the CB subunit of crotoxin (CBa_2 _and CBc), isoform CA_2 _of the acidic subunit of crotoxin, and the VRV-PLVIII from *Daboia russelli pulchella *venom were purified in our laboratory as described previously [55,56,62,67]. CBc was reduced by dithiothreitol and alkylated with iodoacetic acid according to the procedure described by Faure *et al.*[56]. Drs. I. Krizaj (Institute Jozef Stefan, Ljubliana), E. Myatt (Argon Institute), Y. C. Chen (Institut of Biochemistry, Shanghai, China), and J. Perales (Fundaçao Oswaldo Cruz, Rio de Janeiro, Brazil) provided PLA_2 _from *Vipera berus berus*, PLA_2 _from *Crotalus atrox*, AGTX and the basic PLA_2 _from *Agkistrodon hallys pallas*, and MtxII from *Bothrops asper*, respectively. We purchased human activated blood coagulation factor (FXa) from Enzyme Res. Laboratories, USA (MW 46 kDa). The corresponding amino acid sequence shows a Glu residue at position 150 of the heavy chain, whereas UniProt's entry P00742 (FA10_HUMAN) shows an Arg. This residue is at the surface of the molecule. For the simulations, we chose the 2.2 Å resolution crystallographic structure of Gla-domainless FXa from entry 2BOH [90] of the PDB, which corresponds to sequence FA10_HUMAN and is made up of the heavy chain and of the EGF-like 1 and 2 domains of the light chain. Only light chain residues Arg86-Glu138, which represent the EGF-like 2 domain, and heavy chain residues Ile16-Thr244 are located in the X-ray diffraction experiment -the entire EGF-like 1 domain and its hydrophobic peptide preceding it being disordered. In this structure, FXa is co-crystallized with a 2-carboxyindole inhibitor and a Ca^2+ ^ion chelated by Asp70, Asn72, Gln75 and Glu80 of the catalytic (heavy) chain. We removed the inhibitor and the water molecules for the calculations.

We performed computer graphics using PyMOL (DeLano Scientific LLC, San Francisco, CA, USA), and the insightII and DS Visualizer softwares (Accelrys Inc, San Diego, CA, USA). Computer calculations were performed with the insightII software package on SGI graphic stations and using programs available in servers in the World Wide Web with PC workstations.

### Determination of prothrombinase activity

We determined the anticoagulant potency of the SVPLA_2_s by measuring *in vitro *the inhibition of prothrombinase activity (IC_50_). We used an *in vitro *biological test in which the prothrombinase complex was reconstituted at 37°C from purified human factors FVa and FXa in the presence of Ca^2+^, but without the addition of PL[39]. The purified prothrombinase components (FVa 10 nM, FXa 10 nM, and different concentrations of SVPLA_2_: 0, 10, 20, 50, 100, 200, 500 nM) were incubated 5 min at 37°C in Tris-buffered saline (0.05 M Tris/HCl, 0.1 M NaCl, 0.5% BSA, 5 mM CaCl_2_, pH 7.4). The reaction was then started with 110 nM prothrombin. We measured activated prothrombin activity every 20 min, as described previously [50], with small modifications. We determined the IC_50 _value, which corresponds to 50% inhibition of thrombin generation for the different SVPLA_2_s in the absence of PL.

### Surface Plasmon Resonance studies

We analyzed of the interaction between the SVPLA_2_s from the *Viperidae *family, and FXa by SPR using a BIACORE^® ^2000 system (Biacore AB, Uppsala, Sweden). The running and dilution buffer in all experiments was Hepes (HBS; 10 mM Hepes, 150 mM NaCl, 5 mM CaCl_2_, 0.005% surfactant P20, pH 7.4). The experiments were conducted at 37°C. Human FXa was covalently coupled via primary amino groups on a CM5 sensor chip surface according to Prijatelj *et al.*[39]. One independent flow cell of the same sensor chip was used as a control flow cell and was subjected to a "blank immobilization," i.e., with no FXa added. We found the SPR signal for immobilized FXa on three different flow cells to be 1500 RU, 3000 RU and 5840 RU (1 RU corresponds to 1 pg/mm^2 ^of immobilized protein). We injected PLA_2 _samples (0, 0.25, 0.5, 1, 2, 4, and 8 μg/ml) at 37°C with a flow rate of 20 μl/min on independent runs on the control and assay flow cells and their binding was monitored. Between each injection, we regenerated surfaces with twice 5 μL of 1 M NaCl. The apparent equilibrium constant, <K_d _^app^> = <k_off_>/<k_on_>, the average dissociation rate constant <k_off_> and the average association rate constant <k_on_> were calculated using Biacore's BIAevaluation 3 software. The kinetic models used to fit the data included the Langmuir association, heterogeneous analyte and conformational change. Only the first one showed the lowest closeness-of-fit value (χ^2^). In addition, the other models resulted in affinities much lower (μM) than expected (nM).

### Sequence homologies and alignments

The sequence databases and identifiers are the following: CBc (also known as CB1), Uniprot P62022; CBa_2 _(also known as CB2), P24027; AGTX, P14421; AtxA, P00626; MtxII, P24605; *Vbb*, P31854; CbI, gi 1345182 [57]; CbII, gi 1345181 [57]; CA, isoform CA_2 _[56](obtained by post-translational modification of ProCA, P08878).

For sequence alignments, we used the LALIGN program [91,92], which finds multiple matching sub-segments in two sequences and shows the local sequence alignments. For representing sequences and their alignments, we used Weblogo, a web-based application designed to generate sequence logos [93,94].

### Molecular modeling

Given the high sequence homology between the SVPLA_2_s and diverse PLA_2_s whose crystal structures are available, we applied homology modeling to generate 3D model structures of the SVPLA_2_s using the Biopolymer and Homology modules of the insightII software package (Accelrys Inc. San Diego, CA, USA). We retrieved the PLA_2_s of the template proteins of known 3D structure from the Protein Data Bank[95]. The template proteins are: AGTX, the neutral PLA_2 _from *Agkistrodon halys pallas*[96] (PDB **1A2A**); VRV-PLVIII, the PLA_2 _from *Daboia russelli pulchella*[97] (PDB **1FB2**); MtxII, myotoxin II, a K49-PLA_2 _from *Bothrops asper*. [98] (PDB **1CLP**); the PLA_2 _from *Crotalus atrox*[99] (PDB **1PP2**); the PLA_2 _from *Vipera russelli russelli*[52] (PDB **1VIP**) and *bAhp*, the basic PLA_2 _from *Agkistrodon halys pallas *[100] (PDB **1JIA**).

After building the structurally conserved regions (SCR) and the structurally variable regions (SVR), we replaced the side chains of the template protein by the target protein's side chains in a predetermined library conformation. We then performed a rotamer search assignment in order to avoid atomic steric clashes and optimize the inter-residue energies. We included a structurally conserved catalytic water molecule during the modeling. We did not model the Ca^2+ ^ion at the Ca^2+ ^binding site, since it is absent in several X-ray structures. We subjected the obtained models to an overall internal energy minimization using the CFF91 force field. The protonation state of ionizable side chains and of the N- and C-termini was set for pH 7. Atomic partial charges were those of the CFF91 field. We used a distance-dependent dielectric function of 4r. We applied the cell multipole summation method for van der Waals and coulomb interactions. We used none of the cross terms of the force field. We applied the same conditions to the amino acid residues in the crystal structures used. We checked the stereo chemical quality of the models with the Struct_Check program, and the correctness of the folding with the Profiles3D Verify functionality (self-compatibility scores, insightII). The amino acid residue numbering is based on that of Renetseder *et al.*[15].

### Molecular Electrostatic Potential

The MEP is generated by the combined presence of all partial charges residing on the atoms as a function of their positions. The potential was calculated with the DelPhi 2.5 program in insightII. The grid resolution was of 0.833 Å/grid point. The interior and exterior dielectric constants were 2 and 80, respectively. The value of the ionic strength was 0.145; the probe radius was 1.4 Å and the ionic radius 2.00 Å. The treatment of the grid points at the boundary used a full Coulomb approximation. The values of the potential are given in kT/e units at T = 298 K.

### Molecular docking. Protein interfaces and interface contacts

We generated the molecular complexes with the PatchDock rigid body molecular docking procedure[101]. Given two molecules, the surfaces of the molecules to dock are divided by PatchDock into patches according to the surface shape. These patches correspond to patterns that visually distinguish between puzzle pieces. We used a clustering RMSD criterion of 4.0 Å. The output lists the rank of the complex and its approximate interface area. We selected PatchDock for the docking simulations since it allows *a priori *focusing on the vicinity of potential binding sites. In other words, it is possible to upload a receptor-binding site and a ligand-binding site. We took the SVPLA_2_s as the "receptor" molecules and FXa as the "ligand" molecule.

Thus, we generated molecular complexes only for those SVPLA_2_s for which we find experimental evidence of biophysical interaction, i.e., in which the binding affinity between the PLA_2 _and FXa, as measured by SPR, was high. In addition, we used the available mutagenesis data for AtxA[39] to filter the docked complexes so that AtxA residues that show a decrease in the binding affinity for FXa are at the interface of the complex and define a binding site. The residues defining the binding site on AtxA are Arg72, Lys74, His76 and Arg77 (front edge of the β-wing), and Arg118, Lys127, Lys128 and Lys132 (C-terminal fragment)[39]. We defined no ligand-binding site for FXa.

From the candidate complexes generated by PatchDock for AtxA, only one showed a binding mode compatible with the ensemble of the mutagenesis data -many complexes showed binding to either the β-wing or the C-terminal fragment of the SVPLA_2 _or other regions of the AtxA. Thereafter, we selected those complexes for the other SVPLA_2_s that showed the same binding mode as AtxA and whose Cα RMSDs with respect to the AtxA-FXa complex were minimal. After generation of the complexes, we used the "move apart" option of PatchDock, which separates (by 1.6 Å) the receptor and ligand subunits in order to eliminate steric hindrances at the interface. We further improved the fitting of the complex by applying firstly the SCWRL3 side chain modeling procedure [102], in which we froze all disulfide bonds, as well as the side chains of heavy chain residues Asp70 and Glu80, which chelate the Ca^2+ ^ion in FXa. After the rotamer search, we applied additional energy minimizations in order to reach a minimal internal energy conformation. The entire approach assumes that no drastic conformational changes occur during complexation.

On another hand, we submitted the SVPLA_2 _models to the Protein-Protein Interface Prediction (PPI-Pred) server [26,103] to predict their binding sites. PPI-Pred predicts protein-protein binding sites using a combination of surface patch analysis and a support vector machine trained on 180 proteins involved in both obligate and non-obligate interactions.

The interface between the two polypeptide chains of each of the complexes was characterized with the Protein interfaces, surfaces and assemblies service PISA [104,105]. The interface contacts were obtained through a contact map analysis and characterized with the SPACE bioinformatics tools CMA (Contact Map Analysis) and CSU (Contacts of Structural Units) [106,107]. We show only interface contacts for which the contact area is equal to or greater than 10 Å^2^.

## Abbreviations

AGTX: agkistrodotoxin, the neurotoxic, neutral PLA_2 _from *Agkistrodon halys pallas *venom 

*bAhp*: the basic PLA_2 _from *Gloydious *(*Agkistrodon) halys pallas *venom

AtxA: isoform A of ammodytoxin from *Vipera ammodytes ammodytes *venom

CA_2_: one of the isoforms of the acidic subunit of crotoxin

CBa_2_, CBc: isoforms of the basic subunit of crotoxin

CbI: the α isoform of the acidic subunit of the CbI-CbII complex from *Pseudocerastes fieldi *venom

CbII: the basic subunit of the CbI-CbII complex from *Pseudocerastes fieldi *venom

CTX: crotoxin, β-neurotoxin from *Crotalus durissus terrificus *venom, made of acidic CA and basic CB subunits

CbI-CbII: β-neurotoxin from *Pseudocerastes fieldii *venom, composed of CbI and CbII subunits

FVa: Activated human coagulation factor V

FXa: Activated human coagulation factor X, also known as Stuart factor or Stuart-Prower factor

hsPLA_2_: Non-pancreatic secreted human group IIA phospholipase A_2_

IBS: Interfacial Binding Site

<k_on_>: average association rate constant

<k_off_:>: average dissociation rate constant

<K_d _^app^>: average apparent dissociation constant = <k_off_>/<k_on_>

MtxII: Myotoxin II from *Bothrops asper *venom

PL: Phospholipids

*Vbb*: the PLA_2 _from *Vipera berus berus *venom

*Catx*: the PLA_2 _from *Crotalus atrox *venom

RU: Resonance units

sPLA_2_: Secreted phospholipase A_2_

SPR: Surface plasmon resonance

SVPLA_2_: Group IIA snake venom secreted phospholipase A_2_

VRV-PLVIII the PLA_2 _from *Daboia russelli pulchella *venom

## Authors' contributions

GF and RCM conceived the study, analyzed the results and wrote the manuscript. All authors performed the research. In particular, GF carried out SPR affinity measurements, prothrombinase inhibition experiments, and purification and carboxymethylation of PLA_2_s; VTG assisted in purification of PLA_2 _and participated in SPR studies and discussions; RCM carried out sequence analysis, molecular modeling and docking simulations.
